# Carcinogenesis promotion in oral squamous cell carcinoma: KDM4A complex-mediated gene transcriptional suppression by LEF1

**DOI:** 10.1038/s41419-023-06024-3

**Published:** 2023-08-08

**Authors:** Yiming Hou, Wenqian Yu, Gaoyi Wu, Zhaoling Wang, Shuai Leng, Ming Dong, Na Li, Lei Chen

**Affiliations:** 1 Department of Orthodontics, School and Hospital of Stomatology, Cheeloo College of Medicine, Shandong University & Shandong Key Laboratory of Oral Tissue Regeneration & Shandong Engineering Laboratory for Dental Materials and Oral Tissue Regeneration & Shandong Provincial Clinical Research Center for Oral Diseases, Jinan, Shandong 250012 China; 2 Research Center of Translational Medicine, Central Hospital Affiliated to Shandong First Medical University, Jinan, Shandong 250013 P. R. China; 3 Department of Otolaryngology-Head and Neck Surgery, Shandong Provincial ENT Hospital, Shandong University, Jinan, Shandong 250022 China; 4 Center of Clinical Laboratory, Shandong Second Provincial General Hospital, Jinan, Shandong 250022 China; 5 School of Stomatology, Heilongjiang Key Lab of Oral Biomedicine Materials and Clinical Application & Experimental Center for Stomatology Engineering, Jiamusi University, Jiamusi, Heilongjiang 154007 China; 6 Department of Oral and Maxillofacial Surgery, School and Hospital of Stomatology, Cheeloo College of Medicine, Shandong University & Shandong Key Laboratory of Oral Tissue Regeneration & Shandong Engineering Laboratory for Dental Materials and Oral Tissue Regeneration & Shandong Provincial Clinical Research Center for Oral Diseases, Jinan, Shandong 250012 China

**Keywords:** Oral cancer, Oncogenes, Epigenetics

## Abstract

Oral squamous cell carcinoma (OSCC) is the most prevalent cancer of the mouth, characterised by rapid progression and poor prognosis. Hence, an urgent need exists for the development of predictive targets for early diagnosis, prognosis determination, and clinical therapy. Dysregulation of lymphoid enhancer-binding factor 1 (LEF1), an important transcription factor involved in the Wnt-β-catenin pathway, contributes to the poor prognosis of OSCC. Herein, we aimed to explore the correlation between LEF1 and histone lysine demethylase 4 A (KDM4A). Results show that the KDM4A complex is recruited by LEF1 and specifically binds the *LATS2* promoter region, thereby inhibiting its expression, and consequently promoting cell proliferation and impeding apoptosis in OSCC. We also established NOD/SCID mouse xenograft models using CAL-27 cells to conduct an in vivo analysis of the roles of LEF1 and KDM4A in tumour growth, and our findings show that cells stably suppressing LEF1 or KDM4A have markedly decreased tumour-initiating capacity. Overall, the results of this study demonstrate that LEF1 plays a pivotal role in OSCC development and has potential to serve as a target for early diagnosis and treatment of OSCC.

## Introduction

Oral squamous cell carcinoma (OSCC) is the most prevalent head and neck squamous cell carcinoma (HNSCC), with an annual incidence of approximately 4 per 100,000 [1, 2]. The risk factors of OSCC are complex, and the disease is characterised by a lack of symptoms in the early stage, strong invasiveness, rapid progression, and poor prognosis [3]. Therefore, further elucidation of the molecular mechanisms underlying OSCC carcinogenesis and the identification of predictive biomarkers and therapeutic targets are crucial.

The highly conserved Wnt/β-catenin signalling pathway, which plays a vital role in embryonic development, adult tissue homeostasis, and cancer progression [4], is reportedly activated in OSCC [5]. Lymphoid enhancer-binding factor 1 (LEF1), an important transcription factor involved in the Wnt-β-catenin pathway, activates the transcription of many downstream target genes (such as cyclin D1, prostaglandin-endoperoxide synthase 2, interleukin-6, and large tumour suppressor kinase 2 [LATS2]) by interacting with β-catenin in the nucleus, and independently modulates the activation or repression of transcription [6, 7]. Dysregulation of LEF1 contributes to the progression of multiple cancers, including breast, colon, prostate, and oesophageal squamous cell carcinoma [8–11]. Meanwhile, asporin (ASPN) binds directly to LEF1, promotes LEF1-mediated gene transcription independent of β-catenin, and inhibits cell apoptosis in gastric cancer [7]. Moreover, LEF1 negatively regulates cylindromatosis (CYLD) by binding to the *CYLD* promoter site and inhibiting its transcription [12]. Selenite treatment inhibits LEF1 recruitment to the *CYLD* promoter and reduces the average tumour weight in colorectal cancer [13]. In OSCC, LEF1 is overexpressed in tumour tissues compared to non-tumorous oral mucosa [14]. However, its role in regulating cell proliferation and apoptosis during OSCC progression remains to be explored.

Histone modifications play crucial roles in OSCC pathogenesis and progression through gene silencing or activation [15, 16]. In particular, histone lysine demethylases (KDMs) participate in transcriptional regulation by affecting the methylation of H3K4, H3K9, H3K27, and H3K36, which may lead to changes in the expression of tumour-suppressor genes and proto-oncogenes [17–20]. Indeed, KDMs have been identified as potential prognostic markers and therapeutic targets in OSCC [21]. Lysine-specific demethylase 4A (KDM4A), also known as Jumonji domain-containing protein 2A (JMJD2A), is a member of the KDM family that regulates gene expression via its function as a demethylase and recruits chromatin factors [22, 23]. Dysregulation of KDM4A expression has been linked to various human cancers, including breast, colon, gastric, prostate, and non-small cell lung cancers [24–26]. KDM4A acts as a cofactor of the androgen receptor (AR) and stimulates AR-dependent transcription of proliferative genes via its demethylase activity on H3K9me3 in prostate cancer [27]. As a recruiter of chromatin factors, it interacts with nuclear receptor co-repressor (N-CoR) to repress achaete-scute family bHLH transcription factor 2 (ASCL2) expression [28, 29]. The level of KDM4A is notably higher in oral cancer and primary squamous cell carcinoma tissues than in adjacent epithelium [19, 20]; however, the molecular mechanisms responsible for its overexpression remain unknown.

LEF1 contributes to the recruitment of the histone modification enzyme protein arginine methyltransferase 6 (PRMT6) to the high-mobility group (HMG) domain, thus influencing cellular differentiation and proliferation [30]. Herein, we aimed to elucidate the molecular mechanisms related to LEF1 and KDM4A in OSCC and to identify potential targets for the early diagnosis and treatment of OSCC.

## Results

### Upregulation of LEF1 correlates with OSCC progression

**Fig. 1:**
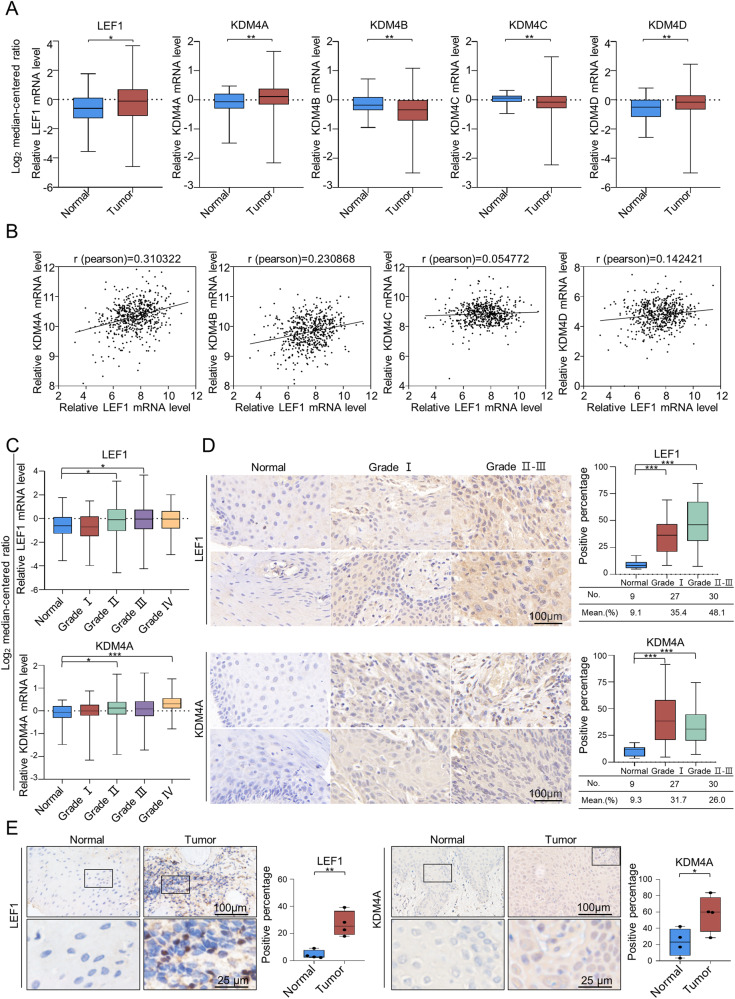
Upregulation of LEF1 correlates with OSCC progression. **A** Analysis of LEF1 and KDM4 family expression in HNSCC using TCGA database. Control: *n* = 44; cancer: *n* = 522. **B** Relative levels of the KDM4 family plotted against LEF1 expression in HNSCC using TCGA database. **C** Analysis of public datasets (TCGA) for the expression of LEF1 and KDM4A in the normal epithelium as well as HNSCCs with histological grades I, II, III, and IV (44 normal, 63 grade I, 305 grade II, 125 grade III, and 29 grade IV). **D** Tissue microarrays showed differential expression of LEF1 and KDM4A in the normal epithelium, histological grade I OSCC, and histological grade II or III OSCC (9 normal, 27 grade I, 30 grade II or III). Positively stained cells (percentage) in the grouped samples were analysed. Scale bar: 100 μm. **E** Immunohistochemical staining of LEF1 and KDM4A in normal epithelial tissues and tumours (*n* = 4). The percentage of positively stained cells (in percentages) in samples were analysed. Scale bar: 100 μm or 25 μm. Data represent the mean ± SD. *p < 0.05, **p < 0.01, ***p < 0.001.

Although LEF1 overexpression has been reported in OSCC [14], its role in OSCC progression remains unknown. Considering that LEF1 is associated with histone modification [30, 31] and that the KDM4 subfamily of histone-modifying enzymes is dysregulated in OSCC [18], we analysed the expression of LEF1 and KDM4s (KDM4A–D) using The Cancer Genome Atlas (TCGA) Head and Neck Cancer dataset. RNA-sequencing (RNA-seq) analysis of 566 samples (44 normal and 522 tumour) suggested that LEF1, KDM4A, and KDM4D were expressed at higher levels in tumour tissues than in normal tissues, whereas KDM4B was expressed at lower levels in tumour tissues (Fig. 1A). Moreover, the expression levels of KDM4A and LEF1 were highly correlated in HNSCC based on the Pearson correlation coefficient (Fig. 1B).

We then analysed the expression of LEF1 and KDM4A and their correlation with histological grades of OSCC (Fig. 1C). Comparing the expression in the normal epithelium and tumour tissues from histological grade I, II, III, or IV revealed that higher histological grades corresponded to higher LEF1 and KDM4A mRNA expression. We further validated the abnormal expression of LEF1 and KDM4A in a clinicopathologically relevant context. At the protein level, we compared the expression of LEF1 and KDM4A by immunohistochemical staining of tissue microarrays comprising 57 human pathological OSCC tissues (27 grade I and 30 grade II or III; Fig. 1D) and 9 normal epithelium samples, as well as four additional human OSCC samples and adjacent normal epithelium samples (Fig. 1E). The results further confirmed that LEF1 and KDM4A were notably upregulated in tumours and that the differential expression of LEF1 and KDM4A was related to OSCC histological grade.

### LEF1 physically interacts with KDM4A

**Fig. 2:**
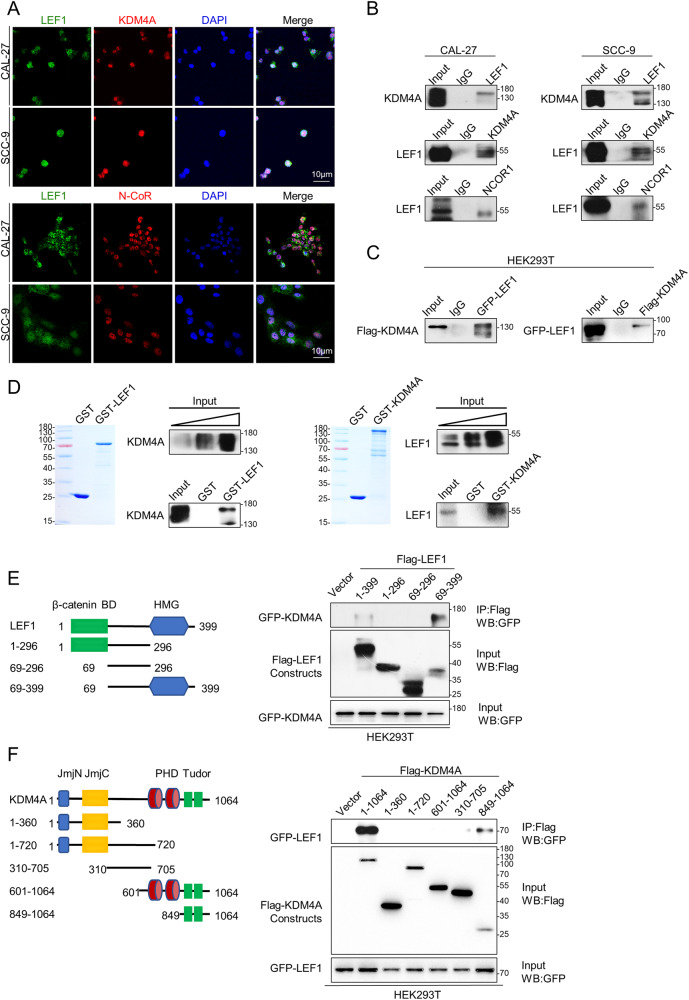
Physically interaction of LEF1 with the KDM4A complex. **A** Normally cultured CAL-27 and SCC-9 cells were fixed and analysed by indirect immunofluorescence using antibodies specific to human LEF1, KDM4A, and N-CoR. Nuclear DNA was stained by 4’,6-diamidino-2-phenylindole (DAPI). Scale bar: 10 μm. **B** Association of LEF1 with KDM4A complex in CAL-27 and SCC-9. Whole-cell lysates were prepared, co-IP was performed using antibodies against LEF1, KDM4A, or N-CoR, and captured samples were immunoblotted with antibodies against the indicated proteins. IgG served as the negative control. **C** Association of LEF1 with KDM4A in HEK293T. Co-IP of exogenous EGFP-tagged LEF1 and FLAG-tagged KDM4A in HEK293T was detected. IgG served as the negative control. **D** GST pull-down experiments performed using bacterially expressed GST-fusion proteins and in vitro-transcribed/translated proteins. **E** Identification of LEF1 domains required for interaction with KDM4A. The Flag-tagged LEF1 or Flag-tagged truncation constructs of LEF1 were overexpressed in HEK293T cells with EGFP-tagged KDM4A. Whole-cell lysates were prepared, Flag and EGFP expression were detected, and co-IP was performed using antibodies against Flag; then, captured samples were immunoblotted with antibodies against EGFP. Cells with overexpression of pCMV6 plasmid and EGFP-tagged LEF1 served as the negative control. **F** Identification of KDM4A domains required for interaction with LEF1. The Flag-tagged KDM4A or Flag-tagged truncation constructs of KDM4A were overexpressed in HEK293T cells with EGFP-tagged LEF1. Co-IP was performed as indicated in **E**.

LEF1 may function by recruiting epigenetic cofactors to target specific genes [30]. To gain a better understanding of the mechanistic relationship between LEF1 and KDM4A, we first analysed LEF1 and KDM4A localisation in two OSCC cell lines, CAL-27 and SCC-9, using immunofluorescence and found that LEF1 colocalised with KDM4A in the nucleus (Fig. 2A). Since KDM4A relies on N-CoR for transcriptional repression [28], we also evaluated whether LEF1 interacted with the KDM4A complex (KDM4A and N-CoR) and found that LEF1 colocalised with N-CoR in the nucleus (Fig. 2A). To reveal their physical association, total proteins from CAL-27 and SCC-9 cells were extracted and co-immunoprecipitation (co-IP) assays were performed. Immunoprecipitants with antibodies against LEF1 proteins were subjected to western blotting (WB) with antibodies against KDM4A, showing that LEF1 physically interacted with KDM4A (Fig. 2B). Similarly, immunoprecipitants with antibodies against KDM4A or N-CoR followed by WB with antibodies against LEF1 confirmed this interaction (Fig. 2B). The interaction between LEF1 and KDM4A was also demonstrated in HEK293T cells overexpressing Flag-KDM4A and GFP-LEF1 (Fig. 2C). We then assessed the LEF1 and KDM4A combination in vitro; reciprocal Glutathione-S-transferase (GST) pull-down was performed using GST-fused LEF1 or KDM4A and in vitro-transcribed/translated KDM4A or LEF1, revealing that the LEF1 and KDM4A molecules interacted directly (Fig. 2D). Collectively, these results suggest an interaction between LEF1 and the KDM4A complex.

Next, we sought to gain insights into the essential domains required for the interaction between LEF1 and KDM4A. LEF1 contains a β-catenin-binding site located at amino acid residues 1–62 and a DNA-binding HMG domain at the C-terminus (residues 299–367; Fig. 2E) [6]. KDM4A contains a higher number of structural domains, including the JmjN domain (residues 14–56), the catalytic JmjC domain (residues 142–308), two plant homeodomain-type zinc fingers (residues 709–767 and 828–885), and two Tudor domains near the C-terminus (residues 897–954 and 955–1011; Fig. 2F) [32]. Co-IP was further performed with various plasmids in HEK293T cells, and proteins were subjected to WB with antibodies against EGFP (enhanced green fluorescent protein). Depletion of the LEF1 β-catenin-binding site did not interrupt KDM4A binding, and expression of the HMG domain resulted in KDM4A interaction (Fig. 2E), indicating the function of this domain goes beyond DNA-binding [6]. Similarly, the Tudor domain of KDM4A not only serves as a reader module that recognises and binds to methylated amino acid residues [33] but is also responsible for the interaction with LEF1 (Fig. 2F). In summary, we verified the interaction between LEF1 and the KDM4A complex and identified its interaction with the HMG domain of LEF1 and the Tudor domain of KDM4A (Fig. 2F). Therefore, we believe that LEF1 can guide the recruitment of the KDM4A complex to the chromatin in OSCC.

### Knockdown of LEF1 or KDM4A suppresses proliferation while promoting CAL-27 and SCC-9 cell apoptosis in vitro

**Fig. 3:**
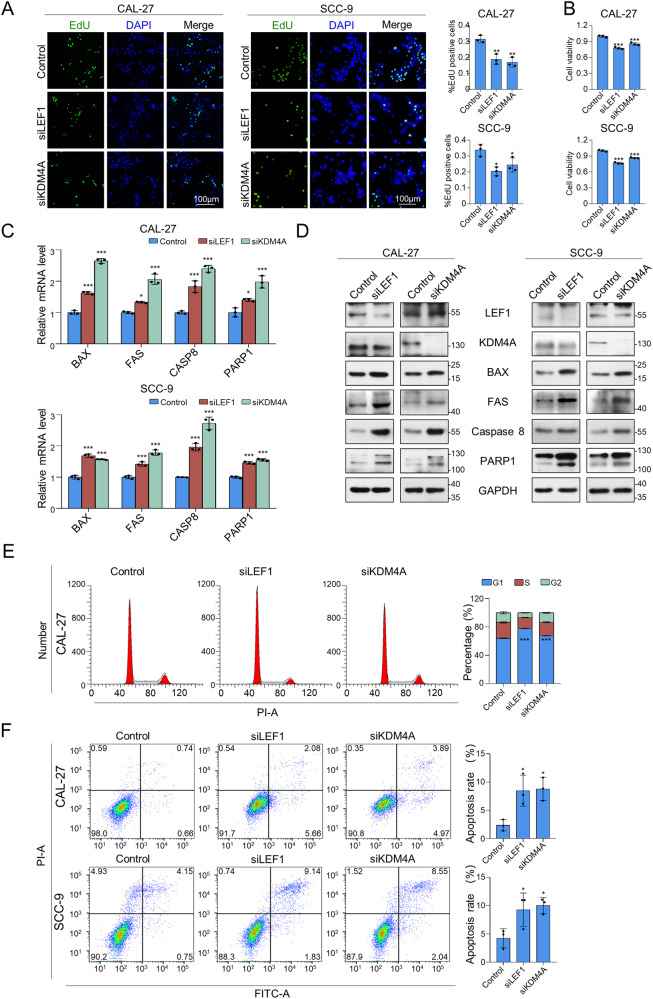
Knockdown of LEF1 or KDM4A suppresses proliferation but promotes apoptosis in CAL-27 and SCC-9 cells in vitro. **A** Twenty-four hour after transfection of LEF1 or KDM4A siRNA, the CAL-27 or SCC-9 cells were incubated with EdU for 3 h. A fluorescence microscope was used to detect EdU. Scale bar: 100 μm. B Cell viability of CAL-27 and SCC-9 48 h after transfection, as assessed by CCK-8. **C** Expression of apoptosis markers measured using RT-PCR in LEF1/KDM4A-depleted CAL-27 and SCC-9 cells. Results are expressed as fold-change relative to control, and *GAPDH* was used as negative control. **D** Expression of apoptosis markers was measured using western blotting (WB) in LEF1/KDM4A-depleted CAL-27 and SCC-9 cells. GAPDH served as a loading control. **E** Apoptosis rate of LEF1/KDM4A-depleted CAL-27 cells assessed by flow cytometry. **F** Cell cycle analysis of LEF1/KDM4A-depleted CAL-27 cells assessed by flow cytometry. Data are shown as means ± SD from three independent experiments. *p < 0.05, **p < 0.01, ***p < 0.001.

Abnormal expression of LEF1 has been implicated in the proliferation and apoptosis of several cancer cells, and LEF1-mediated gene transcription may go beyond the Wnt-β-catenin pathway [7, 34]. Therefore, we investigated the role of LEF1 and KDM4A in the proliferation and apoptosis of oral squamous cells. First, 5-ethynyl-2′-deoxyuridine (EdU) staining revealed that LEF1 or KDM4A knockdown was associated with a notable decrease in the proportion of EdU-labelled cells (Fig. 3A), indicating impaired DNA replication. The Cell Counting Kit-8 (CCK8) results further revealed that cell viability decreased with LEF1 or KDM4A knockdown (Fig. 3B). These findings demonstrated that LEF1 and KDM4A promoted the proliferation of oral squamous cells in vitro.

Next, we measured the expression of apoptosis markers in CAL-27 and SCC-9 cells after LEF1 or KDM4A knockdown. KDM4A or LEF1 knockdown in CAL-27 and SCC-9 cells resulted in increased expression of apoptosis markers, including BAX, FAS, caspase 8, and PARP1, at the mRNA (Fig. 3C) and protein (Fig. 3D) levels. Additionally, flow cytometry showed that depletion of LEF1 or KDM4A induced cell cycle arrest in the G1 phase in CAL-27 cells (Fig. 3E); this depletion contributed to apoptosis (Fig. 3F) in both cell lines. Hence, LEF1 and KDM4A promote proliferation and inhibit apoptosis in vitro, which is potentially associated with the regulation of LEF1/KDM4A target genes.

### Transcriptome identification of the LEF1/KDM4A complex transcription targets

**Fig. 4:**
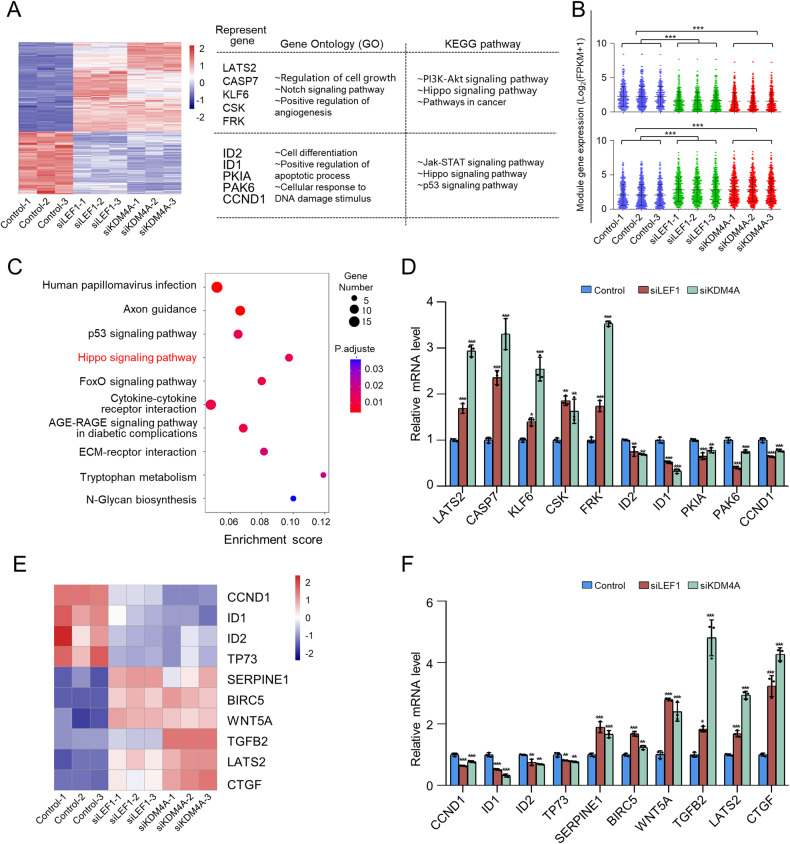
Transcriptome analysis of LEF1 and KDM4A. **A** Heatmap of coregulated gene expression after LEF1/KDM4A knockdown. Representative genes, GO terms, and KEGG pathways of upregulated or downregulated genes are also shown. LEF1 or KDM4A was knocked down in CAL-27 cells by using siRNAs. Three independent samples were separately subjected to RNA-seq analysis. **B** Box plot showing the fragments per kilobase of transcript per million mapped reads (FPKM) of gene expression distribution of upregulated or downregulated genes. For each sample, the target groups were separately compared to three control samples. **C** Pathway analysis of LEF1/KDM4A-regulated target genes arranged into functional groups. **D** Verification of RNA-seq results through qPCR analysis of the indicated genes in CAL-27 cells. Results are represented as fold-change compared to the control, with GAPDH used as the internal reference. **E** Heatmap of differentially expressed genes from the Hippo signalling pathway. **F** Verification of important genes in the Hippo signalling pathway through qPCR analysis in CAL-27 cells. Results are represented as fold-change compared to the control, with GAPDH used as the internal reference. Data represent mean ± SD of three independent experiments. *p < 0.05, **p < 0.01, ***p < 0.001.

To confirm that LEF1 mediates KDM4A recruitment to target genes, we identified genes coregulated by LEF1/KDM4A. Whole-transcriptome clustering analysis revealed that LEF1 knockdown caused the expression of 2468 genes to exhibit a >1.5 fold-change, while KDM4A knockdown induced differential expression of 2478 genes (*Q* value < 0.05). Among these genes, 401 were co-upregulated, and 281 were co-downregulated in the siLEF1 and siKDM4A groups (Fig. 4A–B). The genes coregulated and regulated by LEF1 or KDM4A are presented in Differentially Expressed Genes Identified by RNA-sequencing analysis.

Kyoto Encyclopedia of Genes and Genomes (KEGG) pathway enrichment analysis was performed to further explore the signalling cascades downstream of LEF1 and KDM4A (Fig. 4C). Although knockdown of LEF1 or KDM4A in CAL-27 cells led to the altered expression of several crucial genes at the transcriptional level (Fig. 4D), we focused on the Hippo signalling pathway, which is associated with cell proliferation, cycle arrest, and apoptosis [35]. To explore the importance of this cascade in LEF1/KDM4A regulation, we generated a heatmap showing the involvement of the Hippo signalling pathway in LEF1/KDM4A depletion by analysing ten differentially expressed genes, which were then validated by real-time reverse transcription-polymerase chain reaction **(**RT-PCR; Fig. 4E–F). Based on the results, we propose that LEF1 recruits the KDM4A complex to alter the expression of a series of genes through transcriptional activation or repression.

### The LEF1/KDM4A complex regulates OSCC carcinogenesis by suppressing LATS2 expression

**Fig. 5:**
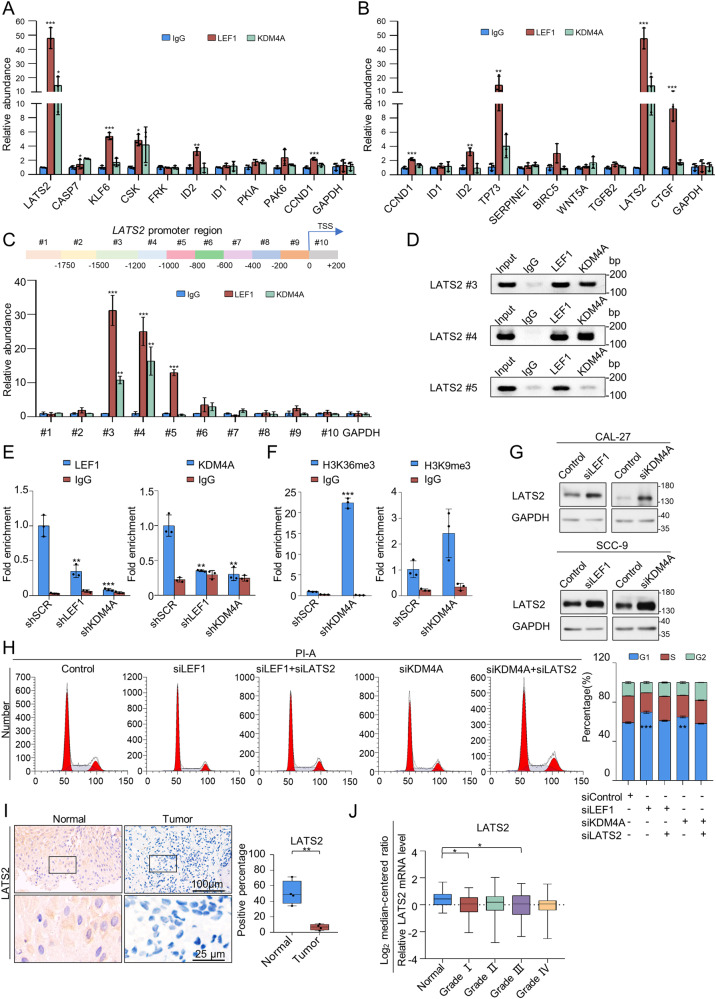
The LEF1/KDM4A complex regulates OSCC carcinogenesis by suppressing LATS2 expression. Verification of RNA-seq results through qChIP analysis of genes in **A** Fig. 4D and **B** Fig. 4F in CAL-27 cells. Results are expressed as fold-change relative to IgG control. GAPDH was used as an internal standard. **C** Primer pairs including #1 to #10 synthesised to cover the promoter region of *LATS2*. qChIP-based promoter-walk experiments were performed using CAL-27 cells; the enrichment of LEF1 or KDM4A was mapped to three or two regions of the LATS2 promoter, respectively. **D** ChIP analysis on CAL-27 cells with antibodies against LEF1 and KDM4A at the *LATS2* promoter. **E** qChIP analysis of LEF1 and KDM4A recruitment to the *LATS2* promoter in CAL-27 cells after transfection with control shRNA (shSCR) or shRNAs targeting LEF1 or KDM4A. IgG served as a negative control. **F** qChIP analysis of H3K9me3 and H3K36me3 enrichment on the *LATS2* promoter in CAL-27 cells after transfection with control shRNA (shSCR) or shRNA targeting KDM4A. IgG served as a negative control. **G** Expression of LATS2 measured using western blotting in LEF1/KDM4A-depleted CAL-27 and SCC-9 cells. GAPDH served as a loading control. **H** CAL-27 cells treated with siRNAs were assessed by flow cytometry to detect cell cycle distribution, revealing that LATS2 knockdown in CAL-27 cells causes altered cell cycle distribution. **I** Immunohistochemical staining of LATS2 in normal epithelial tissue and tumours (*n* = 4). Scale bar: 100 μm or 25 μm. **J** Analysis of public datasets (TCGA) for LATS2 expression in the normal epithelium and HNSCCs with histological grades I, II, III, and IV (44 normal, 63 grade I, 305 grade II, 125 grade III, and 29 grade IV). Data represent the mean ± SD of three independent experiments. *p < 0.05, **p < 0.01, ***p < 0.001.

To explore whether the selected genes are transcriptional targets of LEF1 and KDM4A, chromatin IP (ChIP) experiments were performed in CAL-27 cells. Different levels of LEF1 and KDM4A enrichment were observed in the promoter regions of the selected genes implicated in tumour suppression or promotion (Fig. 5A–B). Among these genes from the Hippo signalling pathway, the DNA segments of the *LATS2* promoter region were highly enriched; *LATS2* encodes a vital serine/threonine protein kinase belonging to the large tumour suppressor family that influences mitosis initiation [36]. Further results showed that the significant enrichments of LEF1 were mapped to three regions of the *LATS2* promoter at upstream ~1500 to ~800 promoter regions. Meanwhile, KDM4A was bound to the upstream ~1500 to ~1000 promoter regions. Accordingly, the upstream ~1500 to ~1000 regions of the *LATS2* promoter might represent the primary region where the LEF1/KDM4A complex negatively regulates *LATS2* expression (Fig. 5C–D). qChIP assays showed that suppression of LEF1 or KDM4A expression resulted in a significant reduction in the recruitment of LEF1 and KDM4A to the *LATS2* promoter. (Fig. 5E). Since KDM4A mediates the demethylation of H3K9 and H3K36, and is more efficient in demethylating tri- versus dimethylated H3K9/H3K36 [32], we analysed the levels of H3K36me3 and H3K9me3 in the *LATS2* promoter of KDM4A-depleted CAL-27 cells. qChIP assays showed that suppression of KDM4A expression resulted in a significant increase in the enrichment of H3K36me3 on the *LATS2* promoter (Fig. 5F).

To better understand the relationship between LEF1, KDM4A, and LATS2, we assessed LATS2 expression following LEF1 or KDM4A knockdown by RT-PCR (Fig. 4D) and WB (Fig. 5G) in CAL-27 and SCC-9 cells. The expression of *LATS2* was markedly increased. We then generated *LATS2*-knockdown CAL-27 cells and conducted a subsequent rescue experiment to determine whether LATS2 is downstream of LEF1/KDM4A. Following LEF1 or KDM4A knockdown, the cell cycle was stalled at the G1 phase. However, in the presence of simultaneous LATS2 knockdown, the proportion of cells in S phase increased (Fig. 5H). Hence, LEF1 might recruit the KDM4A complex to suppress the levels of LATS2, further confirming the role of LEF1 and KDM4A in cell proliferation and apoptosis in vitro.

To extend our observations to a clinicopathological level, immunohistochemical staining was performed to assess LATS2 abundance in the tissues collected from four OSCC patients. LATS2 was expressed at low levels (Fig. 5I). Finally, to verify our observations, we analysed the expression of LATS2 and its correlation with OSCC histological grade in patients using the TCGA Head and Neck Cancer dataset. LATS2 expression decreased with an increase in histological grade (Fig. 5J). These results verified that *LATS2* is negatively regulated by LEF1/KDM4A and the LEF1-KDM4A-LATS2 axis plays a vital role in OSCC.

### LEF1 and KDM4A promote the growth of oral squamous tumour xenografts

**Fig. 6:**
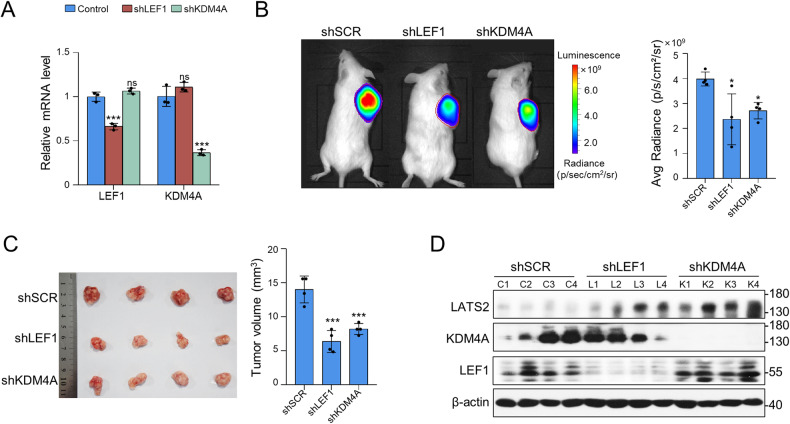
LEF1 and KDM4A promote the growth of oral squamous tumour xenografts. Knockdown efficiency of shRNAs targeting LEF1 or KDM4A. **B** CAL-27 cells infected with lentiviruses carrying the indicated shRNAs were inoculated subcutaneously into the 6-week-old male NOD/SCID mice (*n* = 4). Primary tumours were quantified through bioluminescence imaging at 4 weeks after initial implantation. Representative in vivo bioluminescence images are shown, and tumour specimens were examined using in vitro bioluminescence measurements. **C** Tumour specimens were examined by in vitro measurements. **D** WB was used to verify the efficiency of protein knockdown and the expression of LATS2 in tumours. Data represent the mean ± SD of three independent experiments. *p < 0.05, **p < 0.01, ***p < 0.001.

To investigate the role of LEF1 and KDM4A in oral squamous cell proliferation and apoptosis in vivo, we established mouse xenograft models of CAL-27 cells. The efficiency of the lentivirus-mediated knockdown in CAL-27 cells was verified using RT-PCR (Fig. 6A). Tumour growth was visualised by bioluminescence imaging four weeks after subcutaneous injection (Fig. 6B). Cells stably suppressing LEF1 or KDM4A had markedly decreased tumour-initiating capacity, with tumours from the control shRNA (shSCR) group notably larger than those from the other two groups. The tumour volume of each group was calculated and analysed (Fig. 6C), demonstrating that LEF1/KDM4A promoted the growth of established tumour xenografts. The expression of the indicated proteins in tumour specimens was analysed by WB, revealing higher LATS2 expression in the LEF1- or KDM4A-knockdown groups (Fig. 6D). These experiments verified that LEF1 recruits KDM4A to promote cell proliferation in vivo.

## Discussion

**Fig. 7:**
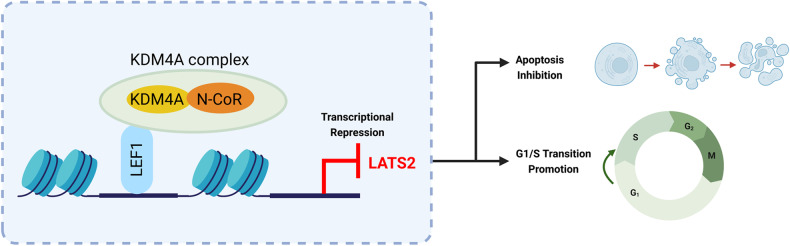
A proposed mechanism of action of the LEF1-KDM4A complex in regulating OSCC cell proliferation and apoptosis. Created with BioRender (https://app.biorender.com).

The transcription factor LEF1 belongs to the T-cell factor (TCF)/LEF family and is known for its role in cancer cell migration, invasion, proliferation, and viability [37, 38]. However, the role of epigenetic modification in LEF1 regulation of cancer progression is poorly understood. Here, we demonstrated that LEF1 expression was positively correlated with that of the histone H3K9/K36 demethylase KDM4A in OSCC. The LEF1-KDM4A complex promotes cell proliferation and inhibits apoptosis by inhibiting LATS2 transcription. Decreased LATS2 expression reverses the phenotypic alteration of OSCC cells induced by LEF1 or KDM4A depletion. Our proposed mechanism of action for the LEF1-KDM4A complex in regulating OSCC cell proliferation and apoptosis in OSCC is shown in Fig. 7.

The Wnt/β-catenin signalling pathway plays a vital role in cancer development [4]. As a key transcription factor of this pathway, LEF1 alters the expression of downstream genes involved in cell proliferation, organ development, and cellular immunity [39]. Indeed, LEF1 functions as an oncogenic molecule in various tumours [9, 11–14]; our results demonstrated that its expression was upregulated in OSCC and promoted cancer in vitro and in vivo. KDM4 family members, serving as histone-modifying enzymes, play a role in transcriptional regulation by interacting with transcription factors. For example, TCF4, another key transcription factor in Wnt signalling cascades, interacts with KDM4C, promoting HP1g removal and transcriptional activation in the nucleus [40]. Among the KDM4 family, KDM4A is the best explored, and its abnormal upregulation is closely related to OSCC [23]. Therefore, we investigated whether LEF1 cooperates with KDM4 family members to regulate OSCC. As LEF1 had the highest correlation coefficient with KDM4A, we focused on elucidating the relationship between this pair. LEF1 promotes the transcription of downstream target genes of the β-catenin in the Wnt/β-catenin signalling pathway [41, 42], and also functions independently of β-catenin with intrinsic histone deacetylase activity or by interacting with histone-modifying enzymes [30, 31]. Here, we found that LEF1 interacts with the KDM4A/N-CoR transcriptional repression complex in a β-catenin-independent manner. These findings suggest that LEF1 is closely associated with histone modification and that its role in OSCC may depend on different interactions with specific molecules.

KDM4A, a member of the JMJD2 family, is dysregulated in several cancer types and plays an important role in proliferation, invasion, and metastasis through complex mechanisms [43–45]. KDM4A primarily demethylates H3K9me2/me3, with a lower rate for H3K36me2/me3. The demethylation of these two distinct methylation markers by KDM4A reflects divergent functions. That is, while a transcriptional repression function is generally assumed for H3K9me3, H3K36me2/3 is associated with active transcription, the detailed mechanism for which remains unclear. Nevertheless, in single-celled organisms, PH domain-containing protein in retina 1 (PHR1) expression is reduced by KDM4A with a lower level of H3K36 methylation at the PHR1 promoter, suggesting that transcriptional repression is strongly influenced by histone demethylase activity [46]. In mammals, enrichment of H3K36me3 represents a universal signature of gene activity, which is correlated with SET domain 2 (SETD2) and recruitment of other chromatin-associated proteins that mediate a range of cellular processes, including transcription elongation, heterochromatin formation, mRNA splicing, and DNA repair [47]. Moreover, enrichment of H3K36me3 at the promoter regions is associated with transcriptional activation. SET and MYND domain-containing Protein 5 (SMYD5), which is recruited to chromatin by RNA polymerase II, acts as a methyltransferase that catalyses H3K36me3, resulting in H3K36me3 enrichment at promoters. In *Smyd5* KO cells, the upregulated genes are associated with high H3K36me3 levels, while the downregulated genes are associated with low H3K36me3 levels [48]. Furthermore, Jie et al. showed that the abnormally high expression of KDM4C in lung cancer inhibits CXCL10 transcription by reducing the enrichment of H3K36me3 at the *CXCL10* promoter region [49]. Transcriptional stimulation or repression of KDM4A may be associated with other molecules, including N-CoR, histone deacetylases, or transcription factors, such as p53 tumour suppressor, in different biological processes [28, 32, 50]. According to our study, KDM4A functions with N-CoR to repress transcription. Evidence from a multicentre cohort tissue microarray study showed that KDM4A could serve as a prognostic marker for OSCC [20]. Further, targeting KDM4A induces DNA replication stress and activates antitumor immunity in HNSCCs, primarily comprising different OSCCs [23]. Our results showed that LEF1 recruits KDM4A and N-CoR, functioning in a coordinated manner to transcriptionally inhibit the tumour-suppressor gene *LATS2* and promote OSCC progression.

LATS2, a serine/threonine kinase in the Hippo signalling pathway, participates in multiple cellular processes, including proliferation, apoptosis, morphogenesis, and differentiation [36, 51, 52]. It plays a critical role in centrosome duplication, mitotic fidelity, and genomic stability and negatively regulates the G1/S transition by reducing cyclin E/CDK2 kinase activity. Here, we observed dysregulation of Hippo following LEF1 and KDM4A knockdown and noticed that LATS2 regulated multiple downstream targets associated with cell cycle and apoptosis [53, 54]. After LEF1 or KDM4A knockdown, the cell cycle was stalled at the G1 phase [18, 55], which may be due to increased LATS2 expression. Meanwhile, qChIP results showed that H3K36me3 levels increased at the *LATS2* promoter with suppression of KDM4A expression. However, H3K9me3 abundance did not markedly change following KDM4A knockdown. Accordingly, we hypothesised that less deposition of H3K9me3 occurred at the *LATS2* promoter in CAL-27 cells. In line with our hypothesis, previous research showed that loss of KDM4A/C mediates an increase in glial fibrillary acidic protein (GFAP) expression, which correlates with increased H3K36me3 and RNA polymerase II recruitment in transcribed regions, as well as the promoter, without impacting H3K9me3 in the GFAP promoter [56]. We suspect that LEF1 recruits KDM4A/N-CoR and may recruit other epigenetic modification complexes to maintain the homeostasis of histone modifications at target promoters, thereby transcriptionally suppressing tumour-suppressor genes such as *LATS2* and inhibiting the progression of OSCC. Additionally, altering the level of KDM4A-induced H3K36me3 demethylation may be a part of the process. However, the specific chromatin factors recruited by the LEF1/KDM4A/N-CoR complex and their functions, and the role of KDM4A as a demethylase in this process, require further investigation.

Although we have proposed a mechanism of action for the LEF1-KDM4A complex in regulating cell proliferation and apoptosis in OSCC, it remains to be explored whether other molecules from the Wnt/β-catenin pathway interact with other KDM family members and if the LEF1-KDM4A complex asserts its effect through altering the methylation level of H3K9 or H3K36.

Certain limitations were noted in this study regarding the clinical samples, particularly those from poorly differentiated neoplasm. Our study emphasises the LEF1-KDM4A axis, while the association of the KDM4 family and Wnt/β-catenin pathway is not fully discussed, and other downstream genes of the axis, save for LATS2 remain to be explored. Moreover, it is unclear whether our findings can be applied to other solid cancers. Finally, the results of this study require validation in clinical settings via the development of agents capable of targeting LEF1-KDM4A-LATS2.

In conclusion, this study revealed that LEF1 recruits KDM4A/N-CoR to suppress LATS2 expression, providing new insights into the functioning of LEF1 in cooperation with histone-modifying enzymes in OSCC. Our data provide a molecular basis for understanding the pathophysiological function of LEF1 and support the hypothesis that the LEF1-KDM4A-LATS2 axis could be a potential therapeutic target for OSCC.

## Materials and methods

An extended description of the used materials as well as tables listing the employed antibodies is provided in Supplementary Fig. and Tables.

### Cell culture and transfection

The CAL-27 cells used in this study were obtained from Procell Life Science & Technology Co., Ltd. (Wuhan, China); the SCC-9 cells were purchased from Shanghai EK-Bioscience Biotechnology Co., Ltd. (Shanghai, China). Both cell lines were STR-authenticated and tested for mycoplasma contamination. CAL-27 cells were cultured in Dulbecco’s modified Eagle’s medium (DMEM; VivaCell, Shanghai, China) supplemented with 10% foetal bovine serum (Gibco, Grand Island, NY, USA) and 1% antibiotics (Gibco). SCC-9 cells were cultured in DMEM/Nutrient Mixture F-12 (DMEM/F12) supplemented with 10% foetal bovine serum, 1% antibiotics, and 400 ng/mL hydrocortisone (Complete Growth Medium from Procell). Small interfering RNA (siRNAs) were transfected at a working concentration of 100 nM using Lipofectamine® RNAiMAX Reagent (Invitrogen, Carlsbad, CA, USA), according to the manufacturer’s instructions. Stable cell lines expressing shLEF1 or shKDM4A were generated by transfecting LV2-shLEF1 or LV2-shKDM4A into CAL-27 cells using the transfection reagent polybrene (Gene Pharma, Shanghai, China), followed by selection with 2 µg/ml puromycin (Sigma-Aldrich, St. Louis, MO, USA). The siRNA and small hairpin RNA (shRNA) sequences designed by Gene Pharma are listed in Table S1 of Supplementary Figures and Tables.

### Patient recruitment

The patients were diagnosed and received treatment at the School and Hospital of Stomatology, Shandong University between May 2021 and July 2022. Patients were included if they had histologically confirmed OSCC and had not receive related treatment prior to tumour removal. Patients were excluded if they had a history of cutaneous HNSCC, a second primary squamous cell carcinoma at a mucosal site outside the oral cavity, or other malignant tumours or serious diseases of vital organs. There were no samples excluded. Four samples of tumour tissue and corresponding adjacent normal tissue, which were confirmed by pathologists, were collected with the permission of the ethics committee of the Stomatological Hospital of Shandong University (No. 20220703) and with patients' informed consent. No blinding method was used for sample collection. Associated clinical information is provided in Table S2 of Supplementary Figures and Tables.

### Immunohistochemistry (IHC) and immunofluorescence staining

For IHC, microarrays of human oral cancer tumour tissues and adjacent normal tissues (HOraC080PG01) were purchased from Shanghai Outdo Biotech Co., Ltd. (Shanghai, China). Pathological information of the tissues is provided in Table S3 of Supplementary Figures and Tables. For the IHC assay, the microarrays or other paraffin section samples were dewaxed with dimethylbenzene and dehydrated using an alcohol gradient (100%, 90%, and 75%). After antigen retrieval with citrate buffer, the samples were blocked with 3% H_2_O_2_ and incubated with donkey serum. Slides were incubated with antibodies overnight at 4 °C as described in Table S4 of Supplementary Figures and Tables, followed by incubation with the appropriate horseradish peroxidase (HRP)-conjugated immunoglobulin G (IgG) polyclonal antibody for 30 min at 37 °C. Diaminobenzidine (DAB) and haematoxylin from Beijing Zhong Shan Golden Bridge Biological Technology Co., Ltd. (Beijing, China) were used to stain the slides. Specimens were assessed based on the positive stained percentage as determined by two independent observers who were not informed of the experiments, using an OLYMPUS-BX530 microscope (Tokyo, Japan).

For immunofluorescence, the cells were fixed with 4% paraformaldehyde for 10 min, permeabilised with 0.2% Triton X-100 in phosphate-buffered saline (PBS) for 10 min and incubated with 0.1% Triton X-100 and 0.4% bovine serum albumin (BSA) in PBS at room temperature. Cells were then incubated at 4 °C overnight with the following primary antibodies: rabbit polyclonal anti-JMJD2A, mouse monoclonal anti-LEF1, or rabbit polyclonal anti-N-CoR. Imaging was performed using a TCS SP8 Leica confocal laser scanning microscope and images were acquired using the Leica Application Suite X (LAS X, Leica, Wetzlar, Germany).

### Proliferation assay and cell viability assay

CAL-27 and SCC-9 cell proliferation was measured with the Click-iT™ EdU Cell Proliferation Kit for Imaging (Invitrogen) and CCK8 (MedChem Express, Monmouth Junction, NJ, USA), according to the manufacturer’s instructions. For the EdU assay, CAL-27 and SCC-9 cells were seeded at a density of 1 × 10^5^ cells/well in 12-well plates with glass slides in each well. Cells were then treated with siRNAs which were diluted to a working concentration of 100 nM and transfected using Lipofectamine® RNAiMAX Reagent (Invitrogen). The medium was replaced with normal medium after 8 h, and the cells were subsequently cultured for an additional 24 h. EdU solution was added to the medium for the last 3 h of the 24 h incubation period. EdU incorporation was detected using a fluorescence microscope (OLYMPUS-BX530).

For the CCK8 assay, 5 × 10^3^ cells treated with siRNAs were seeded in a 96-well plate 2 days before reagent addition, and after a 2 h incubation, proliferation was measured using a colorimetric assay with SpectraMax i3x (Molecular Devices. San Jose, CA, USA).

### Flow cytometry

Cell cycle analysis was performed using a Cell Cycle and Apoptosis Analysis Kit (US Everbright® Inc., Suzhou, China). Briefly, cultured cells were trypsinised to produce single cells, which were then fixed with 70% ethanol at −20 °C overnight. Cells were stained with propidium iodide for cell cycle analysis, which was performed using a cell analyser (BD LSRFortessa, BD Biosciences, San Jose, CA, USA). Data were collected and analysed using ModFit LT software (Verity Software House, ME, USA).

Apoptotic cells were quantified using the Annexin V-FITC Apoptosis Detection Kit (Beyotime, Shanghai, China). Cultured cells were digested with trypsin to produce single cells, which were then stained with annexin V-FITC and propidium iodide, according to the manufacturer’s instructions. Apoptosis analysis was performed using BD LSRFortessa analyser. Data were collected and analysed using FlowJo software (BD Biosciences).

### RNA-sequencing analysis

siRNA was used to knockdown LEF1 and KDM4A expression in CAL-27 cells; three independent samples and controls were used in these experiments. Total RNA was extracted and purified using oligo(dT)-attached magnetic beads, and RNA-sequencing was conducted on an Illumina NovaSeq 6000 (Illumina, San Diego, CA, USA) by Xiuyue Biol (Jinan, China). Differentially expressed genes between each cell group with a *Q* value < 0.05 and fold-change >1.5 were identified. The datasets supporting this research are available in the National Center for Biotechnology Information Gene Expression Omnibus (NCBI GEO) repository with the accession code GSE230372.

### IP and WB

For IP assays, cells were washed twice with cold PBS, and extracts were prepared by incubating cells in lysis buffer (50 mM Tris-HCl, pH 7.4, 150 mM NaCl, 2 mM EDTA, 0.3% NP-40, and protease inhibitor cocktail [MedChem Express]) for 30 min at 4 °C, followed by centrifugation at 12,000 × *g* for 10 min. Next, 500 μg protein samples were incubated with the appropriate primary antibodies or normal rabbit/mouse IgG at 4 °C for 12 h with constant rotation and then mixed with 10 µL Dynabeads Protein G (Invitrogen) for 2 h at 4 °C. After washing the beads three times with cell-lysis buffer (50 mM Tris-HCl, pH = 8.0, 150 mM NaCl, 2 mM EDTA, 0.1% NP-40), the captured immune complexes were stored at −20 °C.

Whole-cell lysates were separated using 8–10% sodium dodecyl sulphate-polyacrylamide gel electrophoresis (SDS-PAGE) Bis-Tris gel, transferred to polyvinylidene fluoride membrane (Merck Millipore, Billerica, MA, USA), blocked in 5% milk, and blotted with antibodies (Table S4 of Supplementary Figures and Tables). The stained bands were detected using enhanced chemiluminescence (Merck Millipore) according to the manufacturer’s instructions.

### GST pull-down experiments

GST-fusion constructs were produced in *Escherichia coli* BL21 cells (Vazyme Biotech Co., Ltd., Nanjing, China), and bacterial lysates were obtained by sonicating the cells at 60 W in cold PBS supplemented with a protease inhibitor (MedChem Express). In vitro transcription and translation experiments were performed using rabbit reticulocyte lysates (Promega, Madison, WI, USA) and plasmid vectors expressing LEF1 or KDM4A according to the manufacturer’s instructions to produce the target protein. For GST pull-down assays, 3–10 μl of the in vitro transcription/translation product was mixed with ~10 μg of the appropriate pGEX-4T-3-fusion protein and incubated in binding buffer (0.8% BSA in PBS containing 1% protease inhibitor). Subsequently, 30 μL of glutathione-agarose beads (GE Healthcare Little Chalfont, Buckinghamshire, UK) were added to the reaction solution and mixed by rotation at 4 °C for 2 h, washed five times with binding buffer, and then resuspended in 30 μl of 2× SDS-PAGE loading buffer. The proteins were detected by WB using specific antibodies.

### LEF1 or KDM4A cDNA expression

LEF1 cDNA (obtained by reverse transcription of CAL 27 cell RNA) and KDM4A cDNA (obtained by reverse transcription of plasmid from Addgene; Cambridge, Massachusetts, USA) were cloned into FLAG-tagged or EGFP-tagged plasmid (Addgene) using restriction endonuclease (Thermo Fisher Scientific, Waltham, MA, USA) and One Step Cloning Kit (Vazyme, Nanjing, China) according to manufacturer’s instructions.

Primers were designed (see Table S5 of Supplementary Figures and Tables) and DNA sequences were synthesised by Sangon Biotech (Shanghai, China). Linearised double-stranded DNA was transformed into competent *E. coli* cells by heat shock and cultured overnight, positive clones were identified by PCR and clone identification was done by the Beijing Genomics Institute (Beijing, China). The sequenced bacterial solution was cultured, and plasmids were extracted using the EndoFree Maxi Plasmid Kit (TIANGEN, Beijing, China). Purified plasmids with the target sequences were transfected into cells using polyethylenimine (PEI, Warrington, Pennsylvania, USA).

### RT-PCR and gel electrophoresis

Total cellular RNA was extracted using TRIzol reagent (Invitrogen) followed by application of chloroform, isopropanol, and 75% ethanol in diethylpyrocarbonate treated water. cDNA was prepared using RevertAid First Strand cDNA Synthesis Kit (Thermo Scientific, MA, USA). PCR amplification was carried out using primers diluted to 10 µM and SYBR Green Real-time PCR Master Mix (TOYOBO, Osaka, Japan), and the thermal cycling protocol was set according to the manufacturer’s instructions: 95 °C for 1 min, followed by 40 cycles of 95 °C for 15 s, 60 °C (or optimised temperature) for 15 s, and 72 °C for 45 s, followed by melting curve analysis. Relative quantitation was performed based on SYBR green fluorescence using an ABI QuantStudio Real-Time PCR System (Applied Biosystems), and the results were obtained using the comparative Ct method (2^-ΔΔCt^) with glyceraldehyde 3-phosphate dehydrogenase (*GAPDH*) as an internal control. The primers used are listed in the Table S6 of Supplementary Figures and Tables.

### Quantitative ChIP (qChIP) assays

qChIP assays were performed using CAL-27 cells as described previously [57]. Briefly, 1 × 10^7^ cells from a 10 cm culture dish were cross-linked with 1% formaldehyde, sonicated, pre-cleared, and incubated with 2 μg of primary antibody against normal rabbit IgG (control), normal mouse IgG (control), LEF1, or KDM4A. The complex was washed with low- and high-salt buffers, and DNA was extracted for qChIP assays. The primers used for qChIP are listed in the Table S7 of Supplementary Figures and Tables.

PCR products were separated by agarose gel electrophoresis with 6× loading dye (Beyotime), and DNA was visualised using an ultraviolet transilluminator (Tanon, Shanghai, China). The primers used are listed in Table S7 of Supplementary Figures and Tables.

### In vivo hyperplasia

Twelve 6-week-old male NOD/SCID mice were purchased from Vital River Laboratories (Beijing, China) and housed at appropriate ventilation conditions with alternating 12 h light/12 h dark cycles at 25 °C. Specific Pathogen Free (SPF) feed and bedding were adopted, and the cages and drinking bottles used were replaced, cleaned, and disinfected every 3 days. The mice were randomly divided into three groups; the four mice in the same group received the same treatment. There were no animals excluded.

CAL-27 cells engineered to stably express firefly luciferase were infected with lentiviruses carrying only the vector, vector + shLEF1, or vector + shKDM4A. The xenograft models were generated via subcutaneous injection of 0.2 ml of CAL-27 cells (5 × 10^6^ cells/0.2 ml) in the right lateral intrascapular areas, and xenograft growth was monitored every 2 days. For bioluminescence imaging, the mice were abdominally injected with 200 mg/g d-luciferin (PerkinElmer, MA, USA) in PBS. Ten minutes after injection, the mice were anaesthetised with gaseous isoflurane (RWD, Shenzhen, China), and a charge-coupled device camera (IVIS kinetic; PerkinElmer) was used to image bioluminescence using the following settings: field of view, D; binning, medium; open emission filter; 4/f stop; and imaging time, auto. Bioluminescence was manually defined based on the relative optical intensity. Photon flux was normalised to the background, defined as the relative light intensity plotted from fluorescein-free mice. After four weeks, the tumours were removed for measurement and WB. No blinding method was used for animal experiments. There were no animal exclusion criteria. Animal handling procedures were approved by the ethics committee of the Hospital of Stomatology, Shandong University (No. 20220704).

### Statistical analysis

Data analysis was performed with GraphPad Prism 8.0 software (GraphPad Software Inc., San Diego, CA, USA). The Shapiro–Wilk test was performed to assess if values fit the normal distribution. Two-tailed unpaired *t*-test or one-way analysis of variance (ANOVA) were used for normally distributed variables. F-testing was used to test homogeneity of variance. If equal variance was not assumed, unpaired *t-*tests with Welch’ s correction or Welch ANOVA tests were used. Differences among non-normally distributed variables were analysed using the Mann–Whitney U-test or Kruskal–Wallis H-test. Correlation coefficients between different genes were calculated using Pearson’s correlation analysis. Results were reported as the mean ± standard deviation (SD) unless otherwise noted. No statistical methods were used to predetermine the sample size. No blinding was used during experiments and outcome analysis. Tumour datasets were downloaded from http://xena.ucsc.edu/, and the number of samples was indicated for each figure.

## Supplementary information

Differentially Expressed Genes Identified by RNA-sequencing analysis

Supplementary Figures and Tables

Original Data File

aj-checklist

## Data Availability

The data generated here are available in the article and its supplementary files. The expression profile data analysed here were obtained from The Cancer Genome Atlas (TCGA) Head and Neck Cancer dataset (https://portal.gdc.cancer.gov/).
