# Supplementary material for: Carcinogenesis promotion in oral squamous cell carcinoma: KDM4A complex-mediated gene transcriptional suppression by LEF1
Source: Cell Death Dis. 2023 Aug 8;14(8):510. doi: 10.1038/s41419-023-06024-3 (PMC10409759; doi:10.1038/s41419-023-06024-3)
Supplement: Supplementary file 2 — Supplementary Figures and Tables [file 41419_2023_6024_MOESM2_ESM.docx]

**Supplementary Information Index:**

1. Supplementary Figures and Figure legends

2- Supplementary Tables

1. **Supplementary Figures and Figure legends**

**Supplementary Figure S1**


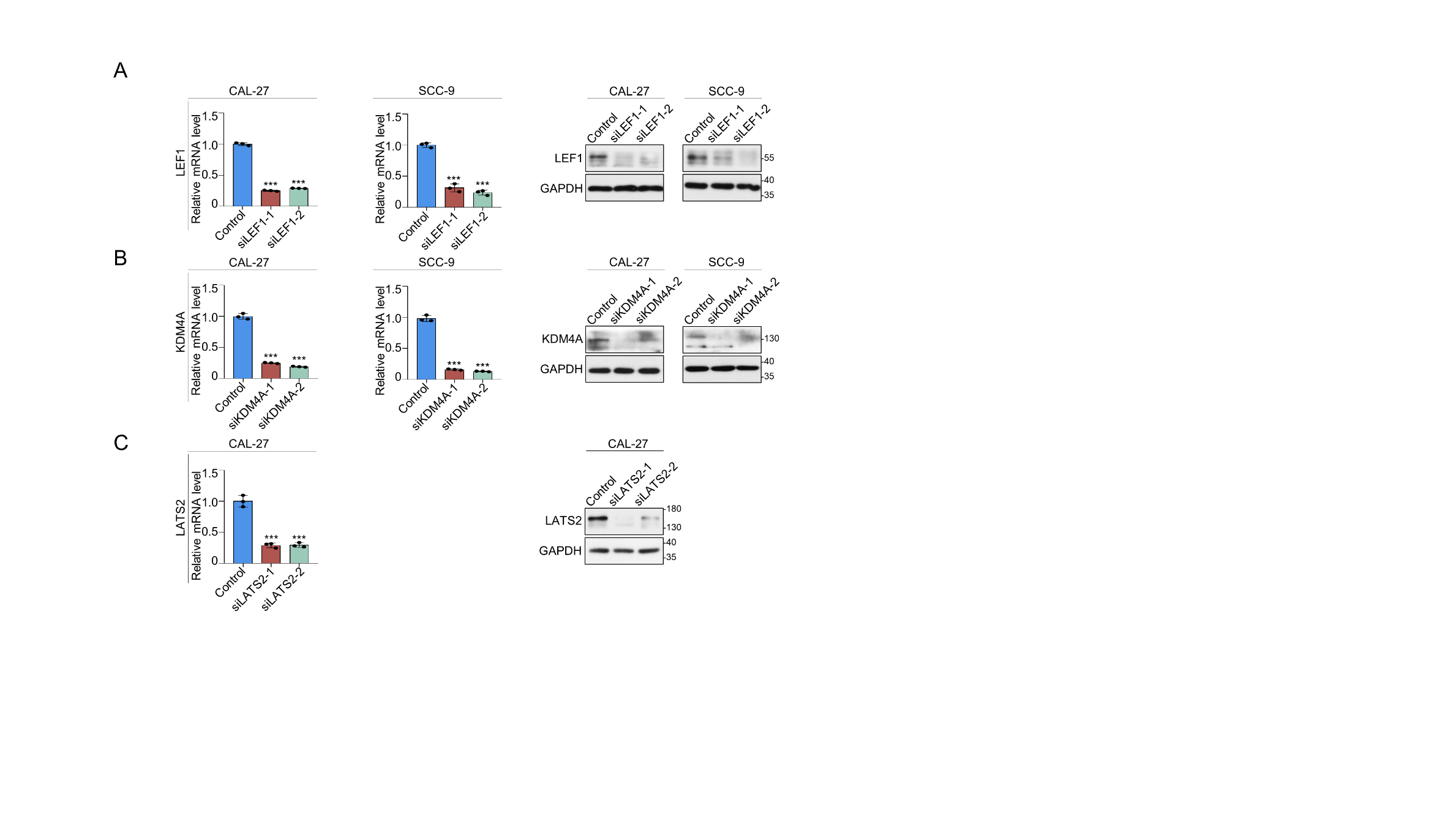


Supplementary Figure S1 RT-qPCR data and western blotting results of the levels of mRNA and protein in LEF1, KDM4A, β-catenin or LATS2 knockdown CAL-27 or SCC-9 cells, the siRNA sequences listed in the supplementary Table S1. A. Knockdown of LEF1. B. Knockdown of KDM4A. C. Knockdown of LATS2. mRNA levels were normalized to those of GAPDH; GAPDH served as a loading control for western blotting. Data represent the mean ± SD of three independent experiments. **p* < 0.05, ***p* < 0.01, ****p* < 0.001.

**2- Supplementary Tables**

Supplementary Table S1. Sequences of siRNA and shRNA.

| Gene | Sequence (5’-3’) |
| --- | --- |
| si-LEF1-01-F | GAGACAAUUAUGGUAAGAATT |
| si-LEF1-01-R | UUCUUACCAUAAUUGUCUCTT |
| si-LEF1-02-F | GUGACCUAAUGCACGUGAATT |
| si-LEF1-02-R | UUCACGUGCAUUAGGUCACTT |
| si-KDM4A-01-F | GCCGUCAGCCUUUAAGCAATT |
| si-KDM4A-01-R | UUGCUUAAAGGCUGACGGCTT |
| si-KDM4A-02-F | GCCUCUUUACUCAGUACAATT |
| si-KDM4A-02-R | UUGUACUGAGUAAAGAGGCTT |
| si-LATS2-01-F | CACGACUUAUUCUGGAAAUTT |
| si-LATS2-01-R | AUUUCCAGAAUAAGUCGUGTT |
| si-LATS2-02-F | CCUACCAGCAGAAGGUUAATT |
| si-LATS2-02-R | UUAACCUUCUGCUGGUAGGTT |
| si-β-catenin-01-F | GCAGCUGGAAUUCUUUCUATT |
| si-β-catenin-01-R | UAGAAAGAAUUCCAGCUGCTT |
| si-β-catenin-02-F | GGACACAGCAGCAAUUUGUTT |
| si-β-catenin-02-R | ACAAAUUGCUGCUGUGUCCTT |
| LV2N-LEF1 | GAGACAATTATGGTAAGAA |

Supplementary Table S2. Patient information of School and Hospital of Stomatology, Shandong University

| Number | Gender | Age（yrs） | Location | Tumor size | Pathological staging |
| --- | --- | --- | --- | --- | --- |
| 1 | male | 63 | palate | 1.5*0.43 | Ⅱ |
| 2 | male | 52 | paradentium | 1.5*2 | Ⅱ |
| 3 | male | 55 | tongue | 1.5*0.6 | Ⅰ-Ⅱ |
| 4 | male | 69 | palate | 2.5*2.5 | Ⅰ-Ⅱ |

Supplementary Table S3. Patient information of HOraC080PG01

| Number | Gender | Age（yrs） | Location | | Tumor size (cm) | Pathological staging |
| --- | --- | --- | --- | --- | --- | --- |
| A01A0006 | female | 64 | paradentium | 2.5*2.5 | | Ⅰ |
| D01A0002 | male | 72 | lip | 2.5*1.5 | | Ⅰ |
| D01A0005 | female | 67 | lip |  | | Ⅱ |
| D01A0006 | female | 76 | lip | 1.1*0.8*0.6 | | Ⅰ |
| D01A0007 | female | 69 | lip | 2*1*1.2 | | Ⅰ |
| D01A0008 | male | unknown | lip |  | | Ⅰ |
| D02A0001 | male | 64 | cheek | 1.5*1 | | Ⅰ |
| D02A0002 | female | 61 | cheek | 3.7*3.5*2.2 | | Ⅰ |
| D02A0005 | male | 58 | cheek | 4.8*2.5*1.2 | | Ⅰ |
| D02A0010 | male | 68 | cheek |  | | Ⅰ-Ⅱ |
| D02A0014 | female | 64 | cheek | 3*2.8*0.7 | | Ⅱ |
| D02A0015 | female | 66 | cheek | 3*2*0.8 | | Ⅰ |
| D02A0016 | female | 72 | cheek | 1.8*1.5*0.3 | | Ⅰ |
| D02A0019 | female | 85 | cheek | 4.5*2.5*1.3 | | Ⅰ |
| D02A0020 | male | 65 | cheek | 1.5*1.2*1 | | Ⅰ |
| D02A0021 | female | 76 | cheek | 1.5*1.2*0.6 | | Ⅰ-Ⅱ |
| D02A0022 | female | 87 | cheek |  | | Ⅲ |
| D02A0023 | male | 59 | cheek | 4*3.5*1 | | Ⅱ |
| D02A0024 | female | 64 | cheek | 2*1*0.8 | | Ⅰ |
| D02A0025 | female | 75 | cheek | 1.8*1.5*0.2；2*1.8*1.5 | | Ⅰ-Ⅱ |
| D02A0027 | female | 74 | cheek |  | | Ⅰ-Ⅲ |
| D02A0028 | male | 77 | cheek |  | | Ⅰ |
| D02A0029 | male | 60 | cheek |  | | Ⅰ-Ⅱ |
| D03A0001 | male | 44 | palate | 4*4*2.5 | | Ⅲ |
| D03A0002 | female | 63 | palate | 2.0*2.0*2.0 | | Ⅰ |
| D03A0004 | female | 66 | palate | 3*2*0 | | Ⅰ-Ⅱ |
| D03A0009 | female | 63 | palate | 2.3*1.8*2 | | —— |
| D03A0010 | female | 67 | palate | 1.2*1*0.6 | | Ⅰ-Ⅱ |
| D03A0011 | female | 69 | palate | 1.5*1*0.6 | | Ⅰ-Ⅱ |
| D03A0012 | male | 67 | palate | 3.5*3.4*1 | | Ⅰ-Ⅱ |
| D03A0014 | male | 45 | palate | 1.5*1*0.2 | | Ⅰ-Ⅱ |
| D03A0015 | male | 54 | palate | 3.5*3*1.3 | | Ⅰ-Ⅱ |
| D04A0001 | female | 63 | paradentium | 4*4*0 | | Ⅱ |
| D04A0009 | male | 58 | paradentium | 9*4*1.8 | | Ⅲ |
| D04A0013 | male | 85 | paradentium | 1.5*1.5*1 | | Ⅱ-Ⅲ |
| D04A0014 | male | 75 | paradentium | 2.1*1.2*0.6 | | Ⅰ-Ⅱ |
| D04A0015 | male | 77 | paradentium | 5*3*1.5 | | Ⅰ-Ⅱ |
| D04A0016 | male | 64 | paradentium |  | | Ⅰ |
| D05A0001 | female | 76 | tongue | 2*1 | | Ⅰ |
| D05A0002 | female | 43 | tongue | 3*2*2 | | Ⅰ-Ⅱ |
| D05A0003 | male | 52 | tongue | 3*2.5 | | Ⅲ |
| D05A0004 | male | 47 | tongue | 3*2*1 | | Ⅱ |
| D05A0006 | female | 62 | tongue | 2*1.5*1 | | Ⅱ |
| D05A0007 | female | 69 | tongue | 4.7*3.2*2 | | Ⅰ |
| D05A0012 | male | 54 | tongue | 4.5*3*1.8 | | Ⅰ |
| D05A0014 | female | 88 | tongue |  | | Ⅱ |
| D05A0015 | female | 81 | tongue | 2.5*1.5*1 | | Ⅰ |
| D05A0016 | female | 80 | tongue | 1.5*1.1*1 | | Ⅰ-Ⅱ |
| D05A0017 | female | 52 | tongue | 1.3*0.8*0.5 | | Ⅰ |
| D05A0018 | female | 53 | tongue | 1.8*1.3*0.7；0.7*0.5*0.5 | | Ⅰ-Ⅲ |
| D05A0019 | male | 80 | tongue | 2.2*1.6*0.4 | | Ⅰ-Ⅱ |
| D05A0021 | male | 57 | tongue | 2*1.5*1 | | Ⅰ-Ⅱ |
| D05A0022 | female | 54 | tongue | 3.5*2.3*2 | | Ⅰ-Ⅱ |
| D05A0023 | male | 50 | tongue | 3*2.5*1 | | Ⅰ |
| D05A0025 | male | 46 | tongue | 1.5*1.5*1 | | Ⅰ |
| D05A0027 | male | 63 | tongue | 3.5*2*2 | | Ⅰ-Ⅱ |
| D05A0029 | female | 51 | tongue |  | | Ⅰ-Ⅱ |

Supplementary Table S4. Antibodies used in this study.

| Antibodies | Company | Cat No. | RRID |
| --- | --- | --- | --- |
| Anti-LEF1 | Santa Cruz | sc-374412 | AB_10991107 |
| Anti-LEF1 | Bethyl | A303-486A | AB_10953689 |
| Anti-JMJD2A | Bethyl | A300-861A | AB_609461 |
| Anti-KDM4A | ABclonal | A7953 | AB_2770069 |
| Anti-N-CoR | Santa Cruz | sc-515934 |  |
| Anti-BAX | Proteintech | 50599-2-Ig | AB_2061561 |
| Anti-Fas | Abcam | ab82419 | AB_1658628 |
| Anti-Caspase-8 | Abcam | ab25901 | AB_448890 |
| Anti-PARP | Cell Signaling Technology | 9542 | AB_2160739 |
| Anti-LATS2 | Abcam | ab243657 |  |
| Anti-GAPDH | Cell Signaling Technology | 5174 | AB_10622025 |
| Anti-β-actin | Cell Signaling Technology | 3700 | AB_2242334 |
| Anti-H3K9me3 | Abcam | 8898 | AB_306848 |
| Anti-H3K36me3 | Abcam | 9050 | AB_306966 |
| Anti-normal mouse antibody | Santa Cruz | sc-2025 | AB_737182 |
| Anti-normal rabbit antibody | Cell Signaling Technology | 2729 | AB_1031062 |
| Peroxidase AffiniPure Goat Anti-Mouse IgG (H+L) | Jackson | 115-035-003 | AB_10015289 |
| Peroxidase AffiniPure Goat Anti-Rabbit IgG (H+L) | Jackson | 111-035-003 | AB_2313567 |
| Anti-rabbit IgG LCS | Abbkine | A25022 | AB_2893334 |
| Anti-mouse IgG LCS | Abbkine | A25012 | AB_2737290 |
| Alexa Fluor 488 donkey anti-rabbit IgG (H+L) | Invitrogen | A21206 | AB_2535792 |
| Alexa Fluor 546 donkey anti-mouse IgG (H+L) | Invitrogen | A10036 | AB_2534012 |

Supplementary Table S5. Sequences of cloning primers.

| PCMV6-LEF1-Hind III-F | GGCGCGCCAGATCTCAAGCTTAAATGCCCCAACTCTCCG |
| --- | --- |
| PCMV6-LEF1-xhol I-R | TTCTGAGATGAGTTTCTGCTCGAGGATGTAGGCAGCTGTCATTCT |
| GFP-LEF1- Xhol I-F | AAGTCCGGACTCAGATCTCGAGCTATGCCCCAACTCTCCG |
| GFP-LEF1- BamH I-R | TTATCTAGATCCGGTGGATCCTCAGATGTAGGCAGCTGTCAT |
| Tag2b-LEF1-BamH I-F | GATAAGAGCCCGGGCGGATCCATGCCCCAACTCTCCG |
| Tag2b-LEF1-Hind III-R | GTCGACGGTATCGATAAGCTTTCAGATGTAGGCAGCTGTCAT |
| Tag2b-LEF1-1-296-BamH I-F | GATAAGAGCCCGGGCGGATCCATGCCCCAACTCTCCG |
| Tag2b-LEF1-1-296-Hind III-R | GTCGACGGTATCGATAAGCTTTCATCTTTTTGGCTCCTGC |
| Tag2b-LEF1-69-296-BamH I-F | GATAAGAGCCCGGGCGGATCCATGGGACACGAGGTGGC |
| Tag2b-LEF1-69-296-Hind III-R | GTCGACGGTATCGATAAGCTTTCATCTTTTTGGCTCCTGC |
| Tag2b-LEF1-69-399-BamH I-F | GATAAGAGCCCGGGCGGATCCATGGGACACGAGGTGGC |
| Tag2b-LEF1-69-399-Hind III-R | GTCGACGGTATCGATAAGCTTTCAGATGTAGGCAGCTGTCAT |
| PCMV6-KDM4A-Hind III-F | GGCGCGCCAGATCTCAAGCTTAAATGGCTTCTGAGTCTGAAACTCTGA |
| PCMV6-KDM4A- xhol I-R | TTCTGAGATGAGTTTCTGCTCGAGCTCCATGATGGCCCGGTATAG |
| GFP-KDM4A- Xhol I-F | AAGTCCGGACTCAGATCTCGAGCTATGGCTTCTGAGTCTGAAACTCTGA |
| GFP-KDM4A- BamH I-R | TTATCTAGATCCGGTGGATCCCTACTCCATGATGGCCCGGT |
| Tag2b-KDM4A-BamH I-F | GATAAGAGCCCGGGCGGATCCATGGCTTCTGAGTCTGAAACTCTGA |
| Tag2b-KDM4A-Hind III-R | GTCGACGGTATCGATAAGCTTCTACTCCATGATGGCCCGGT |
| Tag2b-KDM4A-1-360-BamH I-F | GATAAGAGCCCGGGCGGATCCATGGCTTCTGAGTCTGAAAC |
| Tag2b-KDM4A-1-360-Hind III-R | GTCGACGGTATCGATAAGCTTCTAAGGCAGTTCACTCTCCT |
| Tag2b-KDM4A-1-720-BamH I-F | GATAAGAGCCCGGGCGGATCCATGGCTTCTGAGTCTGAAACT |
| Tag2b-KDM4A-1-720-Hind III-R: | GTCGACGGTATCGATAAGCTTCTAGATGTCCGTGCTGCA |
| Tag2b-KDM4A-310-705-BamH I-F | GATAAGAGCCCGGGCGGATCCATGAAGGACATGGTGAAGATC |
| Tag2b-KDM4A-310-705-Hind III-R | GTCGACGGTATCGATAAGCTTCTACAATGGCTTGGTCCTCT |
| Tag2b-KDM4A-601-1064-BamH I-F | GATAAGAGCCCGGGCGGATCCATGAGCAAGCTCCCCCG |
| Tag2b-KDM4A-601-1064-Hind III-R | GTCGACGGTATCGATAAGCTTCTACTCCATGATGGCCCG |
| Tag2b-KDM4A-849-1064-BamH I-F | GATAAGAGCCCGGGCGGATCCATGCACGGCCGCTGC |
| Tag2b-KDM4A-849-1064-Hind III-R | GTCGACGGTATCGATAAGCTTCTACTCCATGATGGCCCGG |
| GST-LEF1-BamH I-F | GATCTGGTTCCGCGTGGATCCATGCCCCAACTCTCCG |
| GST-LEF1-Xhol I-R | CGATGCGGCCGCTCGAGTCAGATGTAGGCAGCTGTCAT |
| GST-KDM4A-BamH I-F | GATCTGGTTCCGCGTGGATCCATGGCTTCTGAGTCTGAAACTCTGA |
| GST-KDM4A-Xhol I-R | CGATGCGGCCGCTCGAGCTACTCCATGATGGCCCGGT |

Supplementary Table S6. Sequences of PCR primers.

| Gene | primer |
| --- | --- |
| hGAPDH-F | GCACCGTCAAGGCTGAGAAC |
| hGAPDH-R | TGGTGAAGACGCCAGTGGA |
| hLEF1-F | AGAACACCCCGATGACGGA |
| hLEF1-R | GGCATCATTATGTACCCGGAAT |
| h-bcatenin-F | AAAGCGGCTGTTAGTCACTGG |
| h-bcatenin-R | CGAGTCATTGCATACTGTCCAT |
| hKDM4A-F | ATCCCAGTGCTAGGATAATGACC |
| hKDM4A-R | ACTCTTTTGGAGGAACAACCTTG |
| hBAX-HRT-F | CCCGAGAGGTCTTTTTCCGAG |
| hBAX-HRT-R | CCAGCCCATGATGGTTCTGAT |
| hFAS-HRT-F | TCTGGTTCTTACGTCTGTTGC |
| hFAS-HRT-R | CTGTGCAGTCCCTAGCTTTCC |
| hCASP8-HRT-F | TTTCTGCCTACAGGGTCATGC |
| hCASP8-HRT-R | GCTGCTTCTCTCTTTGCTGAA |
| hPARP1-F | CGGAGTCTTCGGATAAGCTCT |
| hPARP1-R | TTTCCATCAAACATGGGCGAC |
| hLATS2-F | ACTTTTCCTGCCACGACTTATTC |
| hLATS2-R | GATGGCTGTTTTAACCCCTCA |
| CASP7-HRT-F | CGGTCCTCGTTTGTACCGTC |
| CASP7-HRT-R | CGCCCATACCTGTCACTTTATCA |
| KLF6-HRT-F | GGCAACAGACCTGCCTAGAG |
| KLF6-HRT-R | CTCCCGAGCCAGAATGATTTT |
| h-CSK-F | AGGACCCCAACTGGTACAAAG |
| h-CSK-R | CGTGGAACCAAGGCATGAG |
| h-FRK-F | CTCTGGGAGTACCTAGAACCC |
| h-FRK-R | AGCCTGGTAATCAAACAAAGCC |
| ID2-HRT-F | AGTCCCGTGAGGTCCGTTAG |
| ID2-HRT-R | AGTCGTTCATGTTGTATAGCAGG |
| ID1-HRT-F | CTGCTCTACGACATGAACGG |
| ID1-HRT-R | GAAGGTCCCTGATGTAGTCGAT |
| PKIA-HRT-F | GCCTTGAAATTAGCAGGTCTTGA |
| PKIA-HRT-R | GCTTCCCCACTTTGTTCTGTAG |
| PAK6-HRT-F | ACCAATAGGCATGGAATGAAGG |
| PAK6-HRT-R | GCGGTCGGAAAGAGGAGTTG |
| CCND1-HRT-F | GCTGCGAAGTGGAAACCATC |
| CCND1-HRT-R | CCTCCTTCTGCACACATTTGAA |
| h-TP73-F | CCACCACTTTGAGGTCACTTT |
| h-TP73-R | CTTCAAGAGCGGGGAGTACG |
| h-SERPINE1-F | ACCGCAACGTGGTTTTCTCA |
| h-SERPINE1-R | TTGAATCCCATAGCTGCTTGAAT |
| h-BIRC5-F | AGGACCACCGCATCTCTACAT |
| h-BIRC5-R | AAGTCTGGCTCGTTCTCAGTG |
| h-WNT5A-F | ATTCTTGGTGGTCGCTAGGTA |
| h-WNT5A-R | CGCCTTCTCCGATGTACTGC |
| h-TGFB2-F | CAGCACACTCGATATGGACCA |
| h-TGFB2-R | CCTCGGGCTCAGGATAGTCT |
| h-CTGF-F | CAGCATGGACGTTCGTCTG |
| h-CTGF-R | AACCACGGTTTGGTCCTTGG |

Supplementary Table S7. Sequences of CHIP-qPCR primers.

| Gene | primer |
| --- | --- |
| qCHIP-hGAPDH-F | AGCCACATCGCTCAGACACC |
| qCHIP-hGAPDH-R | CCCATACGACTGCAAAGACCC |
| qCHIP-hLATS2-1-F | ATCTTTGAAGGCAGAGCAG |
| qCHIP-hLATS2-1-R | TTCTCCACCCCAACCC |
| qCHIP-hLATS2-2-F | GGGTTGGGGTGGAGAAAGTA |
| qCHIP-hLATS2-2-R | GCAGTGGCACAATCATAACTTACT |
| qCHIP-hLATS2-3-F | GAGCCCAAAGAAGAGAGAAA |
| qCHIP-hLATS2-3-R | GTCTCACCCAGCCTTCAGT |
| qCHIP-hLATS2-4-F | AGGGACTGAAGGCTGGGTGA |
| qCHIP-hLATS2-4-R | TGGCAAGTGATGCTGAGGG |
| qCHIP-hLATS2-5-F | GAGGATTTGGCTTCCAGTCTAT |
| qCHIP-hLATS2-5-R | AAGTGAGGTCGTGGGTTTTG |
| qCHIP-hLATS2-6-F | CTAGGCTGGAGTGCAATGATG |
| qCHIP-hLATS2-6-R | GGAGGCTGAGGCAAGAGAAT |
| qCHIP-hLATS2-7-F | ACAGGCATGAGCCACCA |
| qCHIP-hLATS2-7-R | TCGGGAGTTCGAGACCAG |
| qCHIP-hLATS2-8-F | ATTCCACTGTCCCAATAGCC |
| qCHIP-hLATS2-8-R | GAAGGAGCCACCAAGTTACC |
| qCHIP-hLATS2-9-F | CCAGTCAAATAGGTCCGAGA |
| qCHIP-hLATS2-9-R | CGTGCTTCCTGCCTCC |
| qCHIP-hLATS2-10-F | GGAAGATGGAGCAGTCGC |
| qCHIP-hLATS2-10-R | GGGAGACCGGGCACTG |
| qCHIP-hCASP7-F | TCTCCCGTGTTTGACTGACT |
| qCHIP-hCASP7-R | GCTTGCTGGTGGAGATGC |
| qCHIP-hKLF6-F | ACGGGCTCAGGGATGGA |
| qCHIP-hKLF6-R | TCTTCTGCGCCGCTCT |
| qCHIP-hCSK-F | TTCCTTGCCGTTGTCTGTCG |
| qCHIP-hCSK-R | AGCCTGCTCTGGAGTCTATTGG |
| qCHIP-hFRK-F | TGCCAGACTCATTGCCC |
| qCHIP-hFRK-R | TGCTAAACTTTGACCTCCAGAT |
| qCHIP-hID2-F | CTTTCAAGGGCAGTGTATGTA |
| qCHIP-hID2-R | CAGGGTTCCTTTTCAAGAAG |
| qCHIP-hID1-F | GCTGTGGGTCTGGGTTGG |
| qCHIP-hID1-R | GGGAATGCGTTTCTTGCG |
| qCHIP-hPKIA-F | CACCCCACTGTTTCTTTGAGC |
| qCHIP-hPKIA-R | ACCTTTCGCCCTCTCACCTAC |
| qCHIP-hPAK6-F | CTCTCAAAGTGCTGGGATTAC |
| qCHIP-hPAK6-R | GATTGAACGCAGTGTTAGATGT |
| qCHIP-hCCND1-F | TGCCCTCGTGGCGTTCT |
| qCHIP-hCCND1-R | GGGTGAGGTGGAGGTGGCT |
| qCHIP-hTP73-F | ACCTCTGCATGGGATTCGTC |
| qCHIP-hTP73-R | CGCACATCTTCATGGCTCAC |
| qCHIP-hSERPINE1-F | CCACTCCTCATCACTCGCATT |
| qCHIP-hSERPINE1-R | CCTAGATCCCTCACATGAACAGTT |
| qCHIP-hBIRC5-F | TTTGCGAAGGGAAAGGAG |
| qCHIP-hBIRC5-R | CCGTGATAAGAAGACAAGGGA |
| qCHIP-hWNT5A-F | GCACAATCACGCCCACAT |
| qCHIP-hWNT5A-R | CGCAGGCAACTGTTCCA |
| qCHIP-hTGFB2-F | CCACAGCGGTCCTCATTTC |
| qCHIP-hTGFB2-R | TGCCAGCAGATAACATCACG |
| qCHIP-hCTGF-F | TCAGTGGACAGAACAGGGC |
| qCHIP-hCTGF-R | CGCAGTATTTCCAGCACC |
