# Supplementary figures and images for: Carcinogenesis promotion in oral squamous cell carcinoma: KDM4A complex-mediated gene transcriptional suppression by LEF1
Source: Cell Death Dis. 2023 Aug 8;14(8):510. doi: 10.1038/s41419-023-06024-3 (PMC10409759; doi:10.1038/s41419-023-06024-3)

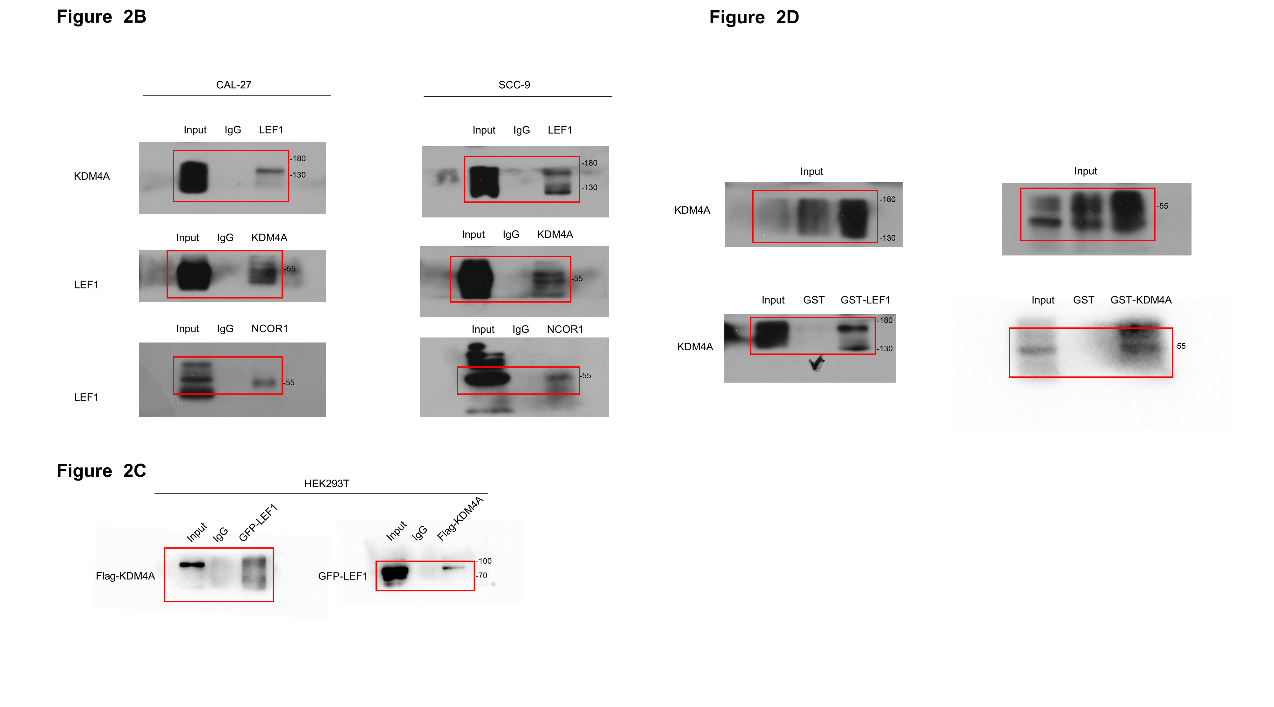

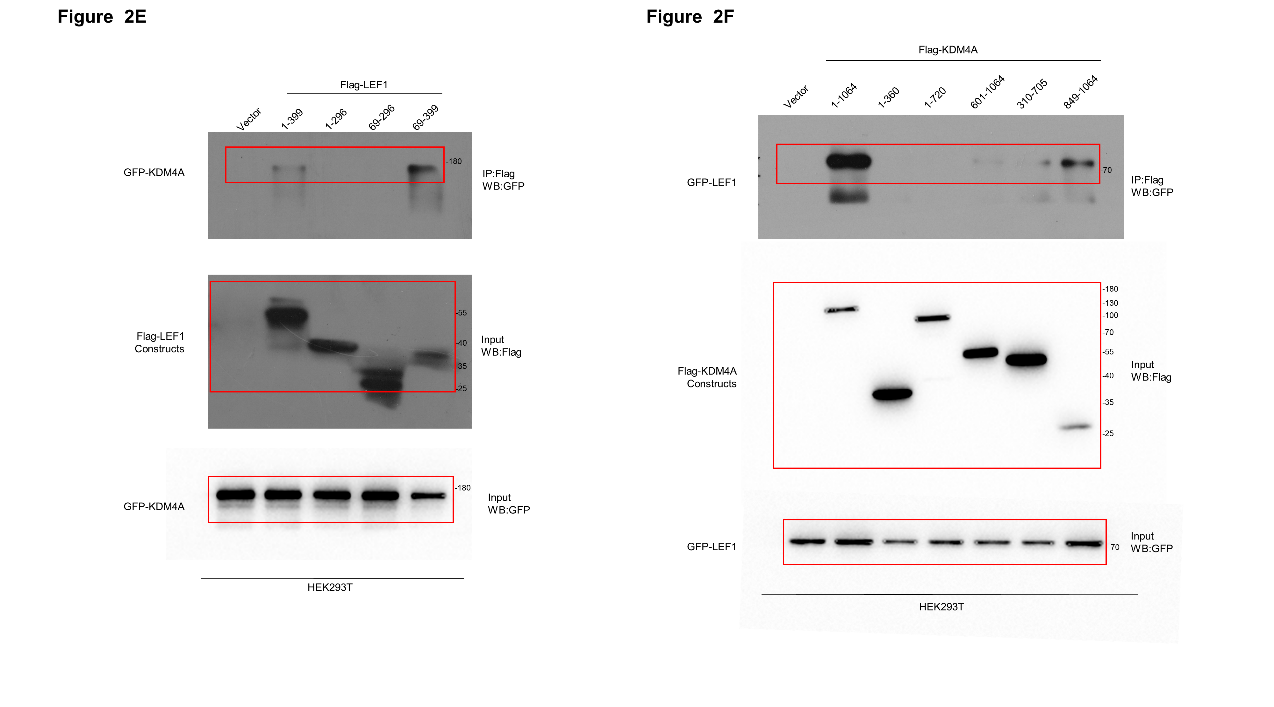

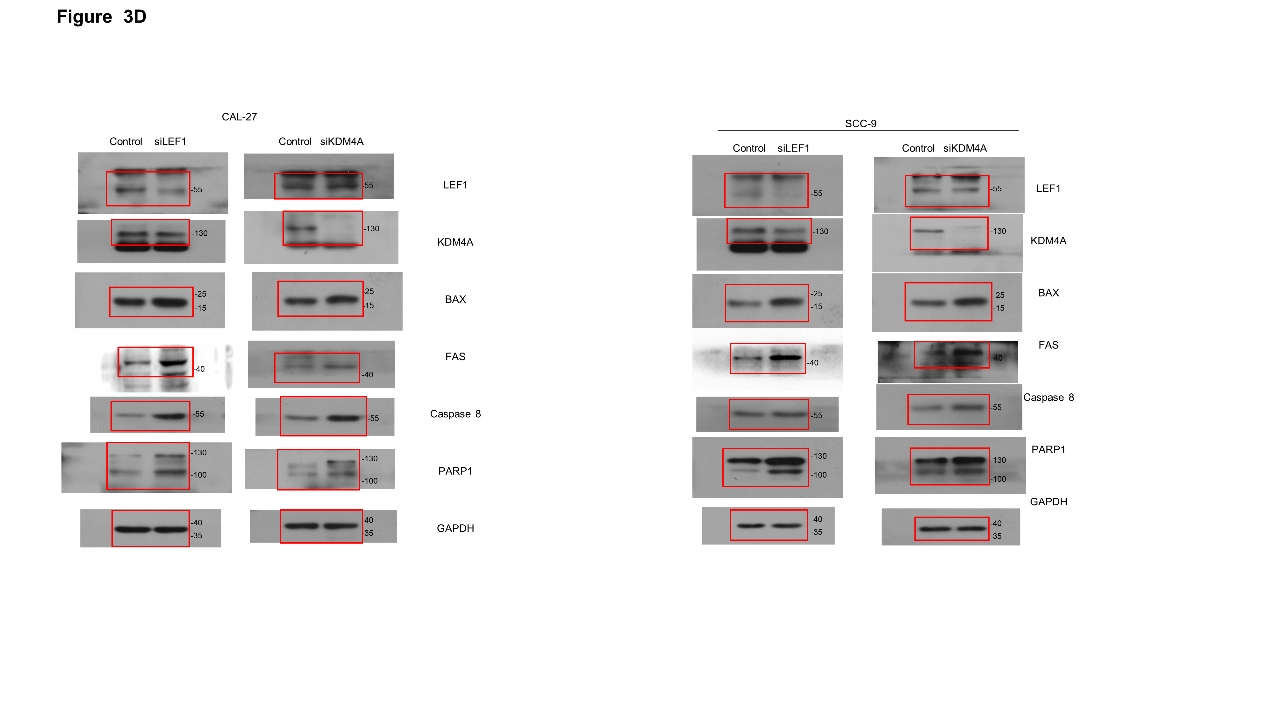

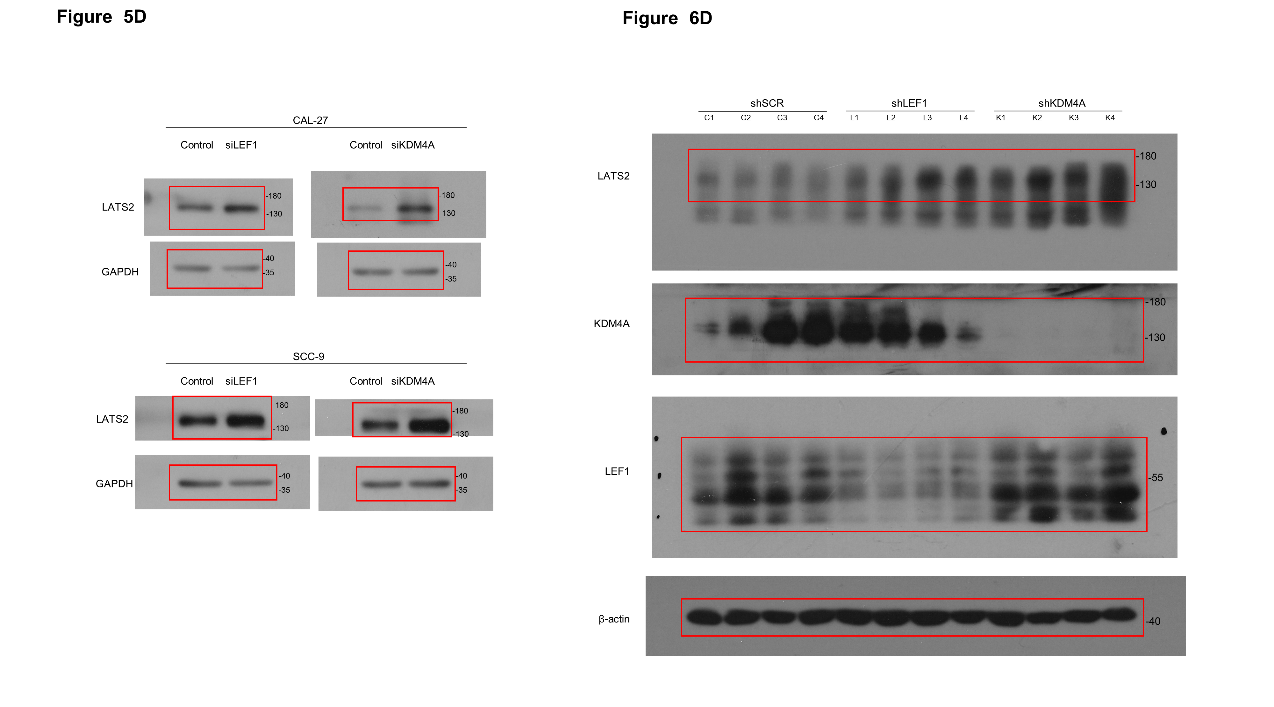

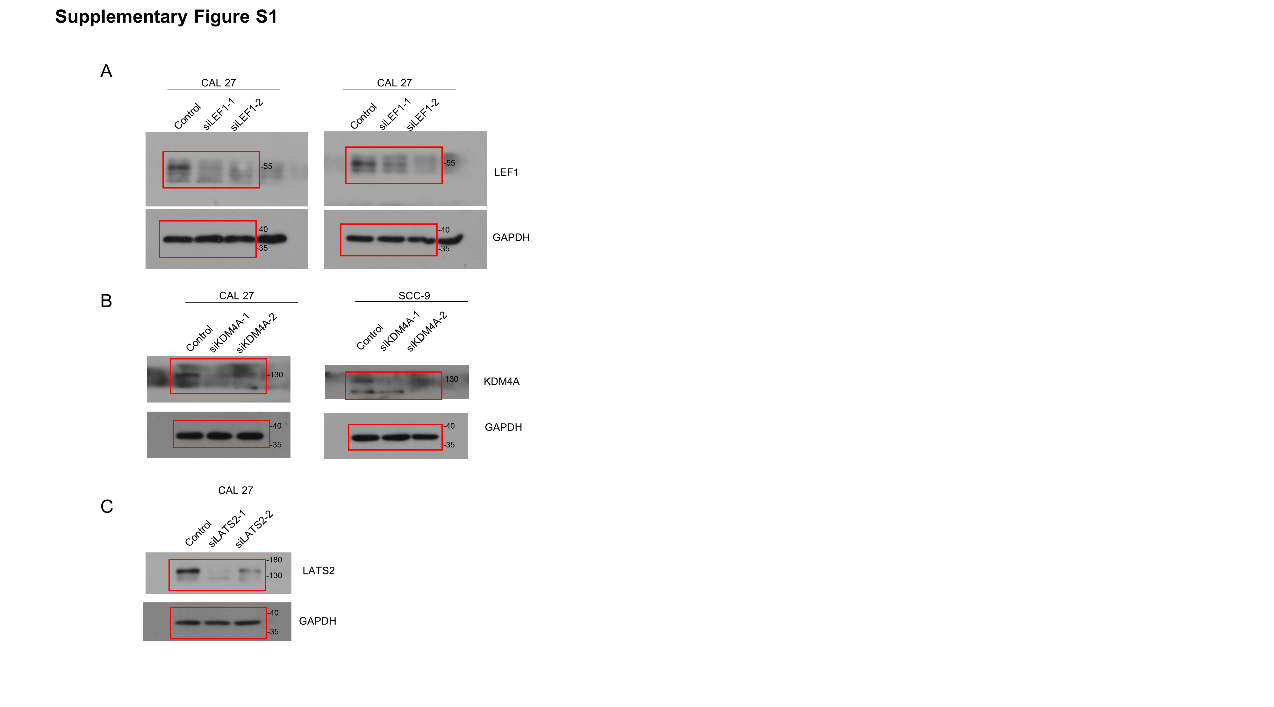

Supplement: Supplementary file 3 — Original Data File [file 41419_2023_6024_MOESM3_ESM.docx]
